# Epidemic Cholera

**Published:** 1857-10

**Authors:** 


*Epidemic Cholera.*

Although this fearful scourge has ceased to manifest itself in any part of our own country, yet it still lingers on the southern border of the continent, as the following paragraph, taken from a reliable source, will show:

*Fearful Ravages of the Cholera in Central America.*—The advices brought from Central America, by the Star of the West, are of a gloomy character. The Asiatic cholera is making sad havoc in Guatemala and San Salvador. The following is an account of its progress in Guatemala:

Hon. W. G. Venable, United States Minister resident to Guatemala, died at the capitol on the 22d, of cholera, which disease he contracted in a few days after he arrived in the country. His remains were interred in the Protestant burying ground. The funeral service of the Protestant Episcopal Church was read by the British Charge d' Affaires, at the grave. His funeral was attended by all the leading officials at Guatemala, and the Diplomatic and Consular representatives of the various foreign powers. Mr. Venable leaves a wife and six children in Tennessee.

The wife of the President of the Republic also fell a victim to the cholera. She died on the 17th of August. The ravages of this scourge throughout Guatemala have been most fearful. It first made its appearance at the capitol on the 8th of July last, and up to the 23d of August there had been 2,494 cases reported, and 1,039 deaths. The smaller towns in the interior have also suffered severely from the epidemic—indeed, the mortality seems to have been greater in several localities than in the capitol. In the town of Villa Nueva, with a population of less than 4,000, some 800 persons have died; and in Amotitlan, with a population of 12,000, it is reported that at least one-twelfth of its inhabitants have died of cholera.

The same account says of the epidemic in San Salvador:

The cholera still continues with unabated violence througho this Republic. Senor San Martin, ex-President of the Repu lic, is among its victims. The number of deaths in the entire State presents a fearful bill of mortality. Up to the 1st of August, it is estimated that between eight and nine thousand persons have died of the disease. The official returns, however, put the number down at only two thousand three hundred and ninety-nine.

